# Observation of Two-Terminal CISS Magnetoresistance with Nonmagnetic Contacts

**DOI:** 10.1021/acs.nanolett.5c01297

**Published:** 2025-06-09

**Authors:** Md Anik Hossain, Sara Illescas-Lopez, Seyedamin Firouzeh, Juan Manuel Cuerva, Luis Álvarez de Cienfuegos, Sandipan Pramanik

**Affiliations:** ◧ Department of Electrical and Computer Engineering, 3158 University of Alberta, Edmonton, Alberta T6G 1H9, Canada; ‡ 16741 Universidad de Granada, Departamento de Química Orgánica, Unidad de Excelencia Química Aplicada a Biomedicina y Medioambiente, C. U. Fuentenueva, Avda. Severo Ochoa s/n, E-18071 Granada, Spain; § Instituto de Investigación Biosanitaria ibs. Avda. De Madrid, 15, E-18016 Granada, Spain

**Keywords:** Chirality induced spin selectivity (CISS), Magnetoresistance (MR), Linear and Nonlinear Magnetotransport, Electromagnetochiral anisotropy (EMChA)

## Abstract

Chiral materials contacted between a ferromagnet (FM) and a nonmagnet (NM) exhibit a chirality-dependent asymmetric magnetoresistance (MR) as the FM magnetization is flipped. This is considered to be a manifestation of the chirality induced spin selectivity (CISS) effect, in which chirality dependent spins generated in the medium are subsequently detected by the ferromagnetic spin detector. Here we show that this two-terminal asymmetric MR response is quite general and can arise even when an *achiral* medium is contacted by *nonmagnetic* electrodes, with no known spin detecting property. By controllably introducing chirality in the channel, we demonstrate how the *chirality-dependent* CISS MR manifests *even for nonspin-detecting electrodes*. The response, distinct from electromagnetochiral anisotropy (EMChA), remains unchanged in the standard configuration with FM/NM electrodes. Thus, observation of chirality-dependent MR in transport experiments does not necessarily relate chirality to spin, as commonly assumed in CISS literature.

## Graphical abstract

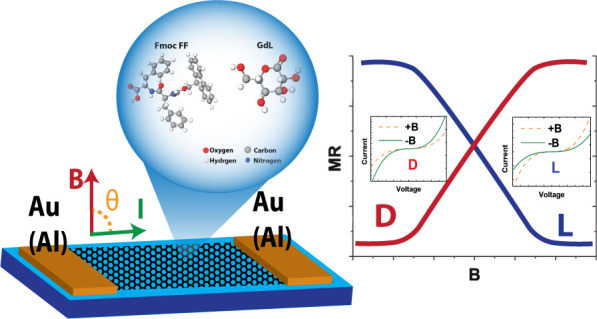

Electronic detection of chirality induced spin selectivity (CISS) is commonly performed using a two-terminal configuration in which a chiral medium is contacted by a ferromagnetic (FM) spin detector and a nonmagnet (NM).^1–3^ According to the standard interpretation, spin unpolarized electrons ejected from the NM acquire a chirality-dependent spin polarization in the chiral medium, and are subsequently detected via the FM spin detector. For a given chirality, the spin polarization is fixed, thus, changing the magnetization of the FM spin detector by an external magnetic field *B* results in different resistance (*R*) values, generating an *asymmetric two-terminal* CISS magnetoresistance (MR): *R*(+*B*) ≠ *R*(−*B*). CISS MR has been reported in many chiral systems^1–3^ using various measurement configurations.^4–13^ Sign of CISS MR correlates with supramolecular chirality of the chiral medium.^11–14^ In addition, current–voltage (*I–V*) characteristics in CISS systems are antisymmetric relative to bias voltage (*V*) *i.e. I*(+*V*) = – *I*(−*V*).

Therefore, based on the standard CISS interpretation, observation of CISS MR requires a spin detecting FM electrode. In contrast, in the present work we show that the CISS MR can manifest even when both contacts are nonmagnetic with no known spin detecting property.

For this purpose, we choose molecular functionalized graphene flakes, which, as shown in our previous work, exhibit all the signatures of CISS MR described above.^15^ Depending on the type of the attached molecule such as Fmoc-GG (Fmoc-diglycine) or Fmoc FF L/D [Fmoc-FF (L): N-Fluorenylmethoxycarbonyl-L-diphenylalanine; Fmoc-FF (D): N-Fluorenylmethoxycarbonyl-D-diphenylalanine], channel chirality can be varied from achiral to L/D chiral, respectively. Figure 1(a) shows the device schematic in which a functionalized graphene thin film, consisting of multiple few-layer (1– 5) graphene flakes (labeled as “SLG” or single layer graphene hereon), is contacted by two planar electrodes (Supporting Information, section I). First, we systematically explore the two-terminal MR of the *achiral* channel with nominally identical nonmagnetic (i.e., nonspin-detecting) contacts with contrasting spin–orbit coupling strengths (such as Au–Au vs. Al–Al) and also with conventional Ni–Au electrodes. Next, chirality is introduced via chiral functionalization and the effect on two-terminal MR is studied. Functionalized graphene flakes typically have a folded structure as shown in Figure 1(a).^15^

**Figure 1:**
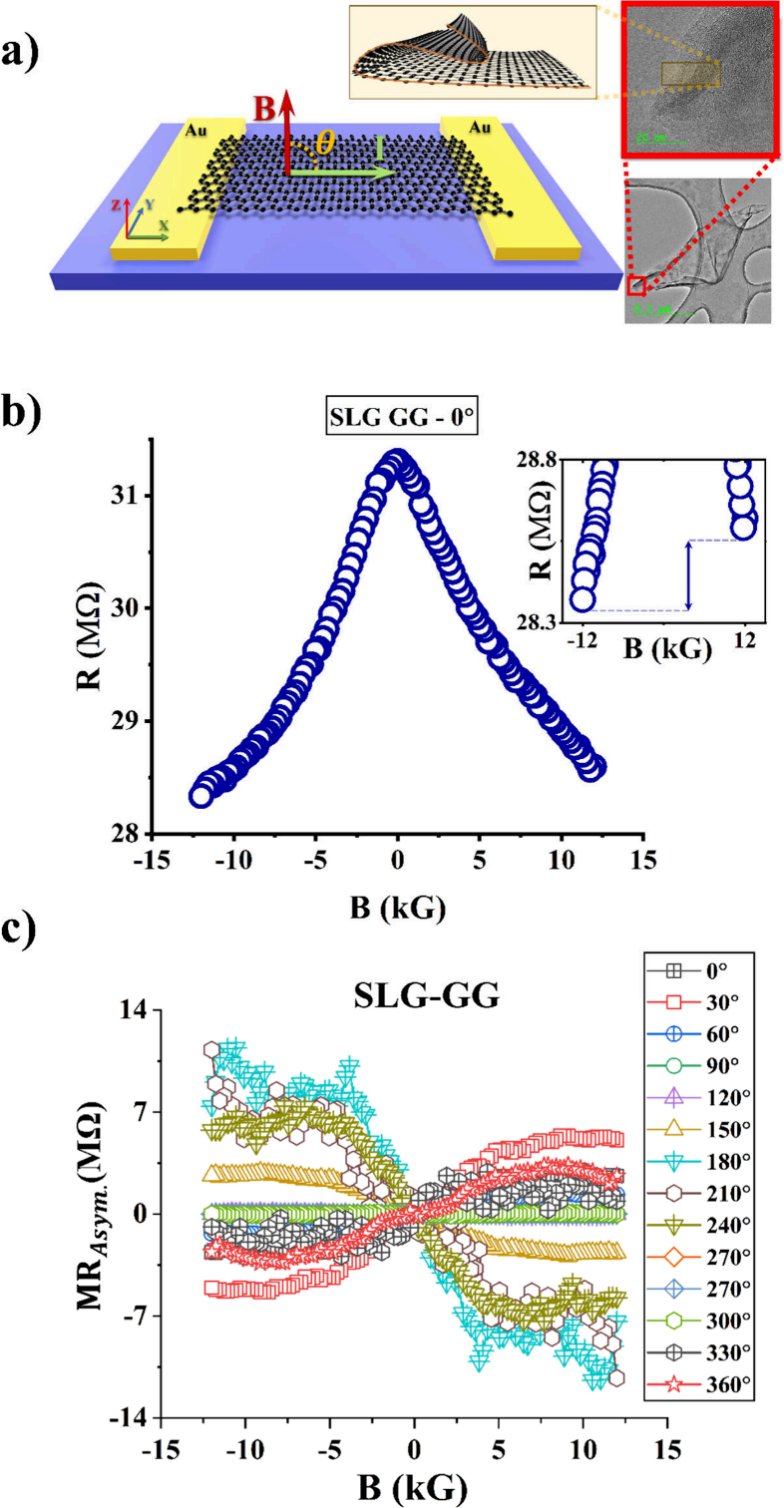
(a) Device schematic and TEM image of a typical folded functionalized graphene flake. Such folded flakes constitute the channel. Chirality of the channel can be tuned via functionalization. (b) Typical asymmetric MR measured with Au–Au electrodes for an achiral (Fmoc-GG functionalized) channel, at θ = 0°. Asymmetric nature of the MR is shown in the *inset*. (c) Asymmetric component of the MR as a function of θ. Measurements in panels b and c have been performed in the nonlinear bias range, with a bias voltage of 0.5 V, as discussed later.

As shown in Figure 1(a), current paths are in the *x-y* plane, and the *average* “drift” current *I* flows perpendicular to the edges of the two contacts. The magnetic field angle (θ) is measured with respect to this direction; θ = 0° when the magnetic field is in-plane and parallel with *I*, and θ = 90°, when the magnetic field is perpendicular to the plane. In the θ = 90° configuration, the magnetic field is perpendicular to *all* current paths in the *x-y* plane.

We start with the simplest configuration in which an *achiral* Fmoc-GG functionalized graphene film is contacted by symmetrical Au–Au electrodes. Absence of chirality is confirmed by the CD signal (Figure S1­(c), Supporting Information). The two-terminal MR at θ = 0° shows a signature global negative MR (Figure 1(b)), consistent with previous reports.^15,16^ Despite the symmetric nature of the contacts and the lack of chirality of the medium, the MR response is found to be *asymmetric*, i.e. *R*(+*B*) ≠ *R*(−*B*) for several field angles θ. The symmetric and asymmetric MR components are computed as MR_Sym_ (*B*) = [*R*(+*B*) + *R*(−*B*)] /2 and MR_Asym_ (*B*) = [*R*(+*B*) – *R*(−*B*)] /2, and an MR “*asymmetry factor*” **χ**_Asym_ is defined as the ratio of the maximum values of these components i.e. **χ**_Asym_ = [MR_Asym_(+12 kG) – MR_Asym_ (−12 kG)]/[MR_Sym_ (0 kG) – MR_Sym_ (±12 kG)] × 100%. The quantity **χ**_Asym_ serves as a normalized parameter for comparison between different types of samples. The asymmetric MR component MR_Asym_ (*B*) is plotted in Figure 1(c) for various θ. Measurements in Figures 1(b) and 1(c) have been made with a bias voltage of 0.5 V, which corresponds to the nonlinear bias range, as discussed later.

The angular dependence of **χ**_Asym_ resembles a ∼ cos θ function as shown in Figure 2(a). The appearance of an asymmetric MR is counterintuitive, because being achiral, Fmoc-GG is not expected to transfer any chirality to the attached graphene channel. To explore the role of any unintentional asymmetry in contact geometry, we tested two different geometries, one with nominally symmetrical contacts, and another one with asymmetric Au–Au contacts. In both cases, nominally identical asymmetric MR responses are obtained (Figure 2(b)). This effectively rules out any role of contact geometry.

**Figure 2:**
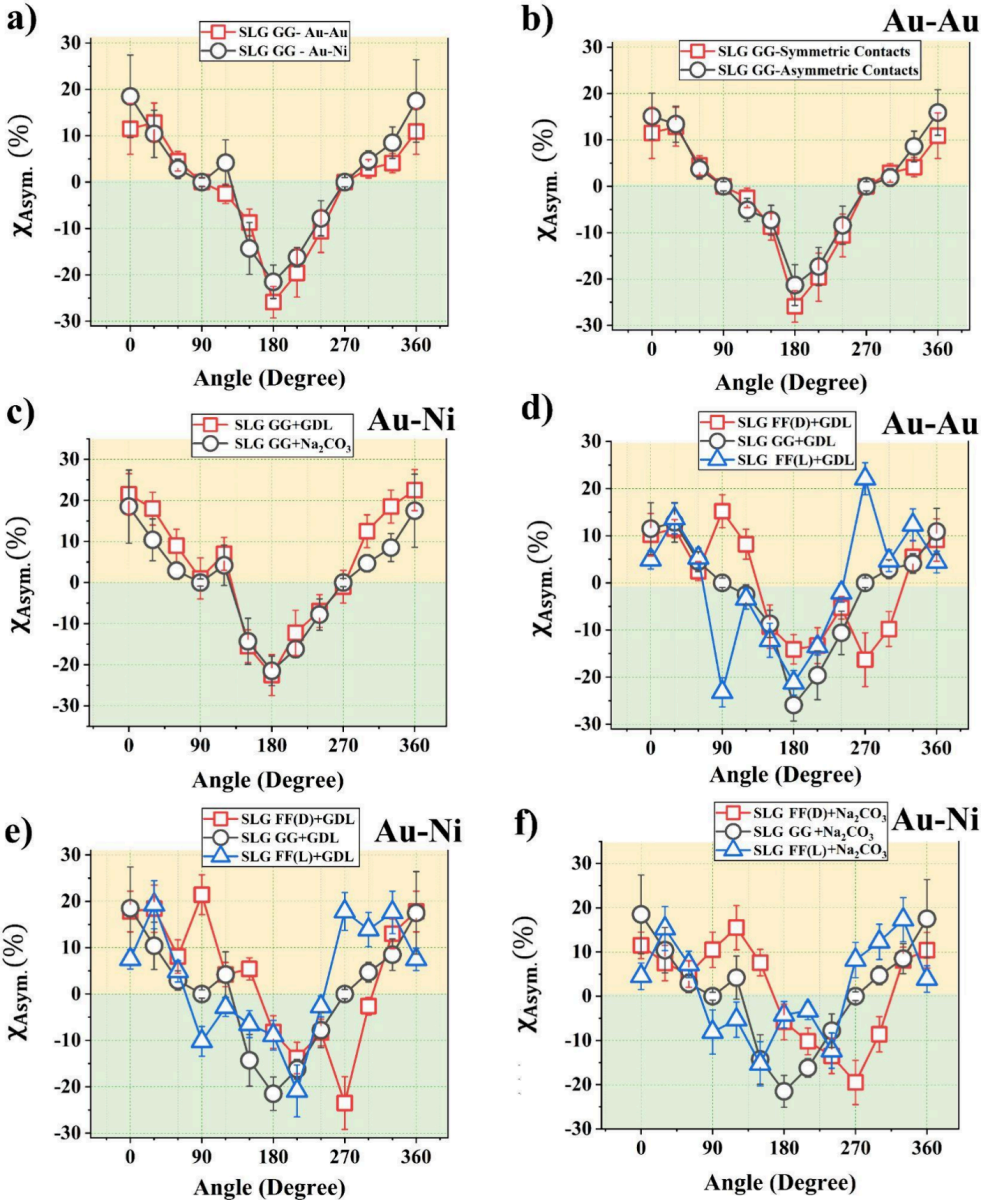
(a) Angular dependence of the MR asymmetry factor **χ**_Asym_ for Fmoc-GG functionalized graphene samples with Au–Au and Au–Ni contacts. Nominally identical asymmetric MR responses, resembling a ∼cos θ behavior, have been observed. (b) Fmoc-GG functionalized graphene samples with symmetric and asymmetric Au–Au contacts. The nominally identical MR responses rule out any potential impact of any contact asymmetry. (c) MR responses from Fmoc-GG functionalized graphene samples synthesized with two different gelling compounds (achiral Na_2_CO_3_ and chiral d-glucono-δ-lactone or GDL), indicating the reproducibility of the results. (d) *Chiral* Fmoc-FF L/D functionalized graphene samples with Au–Au contacts. Chirality dependent signals have been observed at θ ≈ 90° and 270°. The Fmoc-GG signal with Au–Au contacts has been shown for reference. (e) Fmoc-FF L/D+GDL functionalized graphene samples with Au–Ni contacts. Chirality dependent signals have been observed at θ ∼ 90° and 270°. The Fmoc-GG signal with Au–Ni contacts has been shown for reference. (f) Fmoc-FF L/D+Na_2_CO_3_ functionalized graphene samples with Au–Ni contacts. *In all cases the error bars are calculated based on the analysis of two samples per case, each with a minimum of six scans, to confirm the reproducibility of the data.* All measurements have been performed in the nonlinear bias range, with a bias voltage of 0.5 V.

We also tested the Fmoc-GG functionalized SLG samples using standard Au–Ni contacts, common in CISS experiments, which did not change the overall ∼ cos θ response as shown in Figure 2(a). This effect cannot be attributed to any type of anisotropic MR of the contacts, because the overall device resistance is orders of magnitude larger than the resistance of the metallic contacts.

Fmoc-GG functionalized graphene can be synthesized using two different gelling agents – achiral Na_2_CO_3_ and chiral d-glucono-δ-lactone or GDL. Both samples show nominally identical asymmetric MR response with Au–Ni contacts as shown in Figure 2(c), confirming the reproducibility of the measurements. Two-terminal MR of achiral media probed with Au–Ni contacts are rarely reported. Ref 17 reported magneto-conductive AFM measurements of achiral perovskite films, which showed a small MR. This gets enhanced and chirality-dependent when the film is made chiral. Ref 18 observed an asymmetric MR with achiral molecules sandwiched between a ferromagnetic semiconductor and Au electrodes.

Role of spin–orbit coupling of the contacts has sometimes been used to explain the asymmetric CISS MR.^18,19^ To explore this possibility, we perform the angle-dependent MR measurements using Al–Al contacts which have lower spin–orbit coupling strength compared to Au or Ni. Nominally identical results are obtained, as shown in Figure S2.

MR asymmetry can also arise due to the asymmetric shape of the channel.^20^ However, we tested ∼ 10 samples with different channel shapes, but the observed results are remarkably consistent as shown by the error bars in Figures 2 (a)-(c).

Considering Figures 2(a)-(c) and Figure S2, we conclude that an asymmetric two-terminal MR can be present even in nonstandard configurations, where the medium is *achiral* and both the contacts are *nonmagnetic with no known spin detecting property*, and *regardless of their spin–orbit coupling strengths*. The standard CISS interpretation, however, predicts null results in these cases. Ref 21 predicted that achiral systems connected with NM leads can generate spin filtering via breaking of the mirror symmetry between the left/right interfaces. Such absence of interfacial mirror symmetry at a microscopic level is likely in the present case. The lack of chirality also excludes EMChA as a possible mechanism, despite it being independent of the contacts and predicting the ∼ cos θ dependence of **χ**_Asym_ correctly. In addition, there are other qualitative differences between our data and EMChA, as discussed later.

Asymmetric MR in the *nonlinear* transport regime is a well-known phenomenon. Ref 20 observed this effect in asymmetric quantum wires with different sidewall geometries. Ref 22 studied this using a quantum Hall bar with an antidot and a chaotic cavity connected to quantum point contacts, where the asymmetry arises from magnetic-field dependence of the screening potential. Ref 23 reported this effect in single wall carbon nanotubes. In all these cases the contacts are *nonmagnetic*, and the MR asymmetry arises due to *B*-asymmetric *nonlinear* transport and microscopic asymmetry of the samples, *independent* of any spin transport or spin detection.

Similarly, our functionalized graphene samples possess a low degree of symmetry. When achiral Fmoc-GG self-assembles to form a supramolecular structure, the flexible graphene flakes (also achiral) attached to the molecules bend as well (Figure 1(a)). Due to the irregularity of the resulting composite, there exists neither inversion symmetry nor mirror symmetry in the channel, which could cause the observed asymmetric MR similar to the systems discussed above. The bias voltage of 0.5 V used in the measurements of Figure 2 corresponds to the nonlinear range, as discussed later. Now it remains to be seen *whether or how the chirality of the transport medium affects such asymmetric MR*.

Chirality can be induced in the channel by replacing Fmoc-GG with chiral Fmoc-FF L/D (ref CD spectrum in Figure S1 (a)). The measured **χ**_Asym_ for Fmoc-FF L/D functionalized graphene film using *Au–Au* contact pair is shown in Figure 2(d). The previously observed background ∼ cos θ response is still present, *except* in the vicinity of θ ∼ 90° and 270°, where **χ**_Asym_ shows strong deviation from this background, and acquires opposite signs for opposite chiralities. Clearly, this arises due to transfer of chirality from the chiral molecules to the graphene flakes and these MR asymmetries at 90° and 270° mimic CISS MR, due to their chirality dependence. This effect cannot be attributed to EMChA because for θ ∼ 90°, 270°, the chirality-dependent contribution α^L/D^ *B*.*I* ≈ 0. For low symmetry conductors EMChA can be present even for θ ∼ 90°,^24^ however, as discussed later, there are several qualitative differences between our data and EMChA. Further, the background ∼ cos θ signal in this case does not flip sign for opposite chiralities, providing additional evidence on the absence of EMChA.

Finally, we show the angular response of **χ**_Asym_ for Fmoc-FF L/D using standard Au–Ni contacts (Figure 2(e)). The same response observed above appears in this case. The response remains qualitatively unchanged irrespective of the choice of the gelling agent – GDL (Figure 2(e)) or Na_2_CO_3_ (Figure 2(f)) used during functionalization, confirming the reproducibility of the results. All measurements shown in Figure 2 have been done in the nonlinear bias range, with a bias voltage of 0.5 V.

Earlier, for achiral channels, we associated the asymmetric MR with the inherent asymmetry of the channel and nonlinear transport. The results from the chiral channel lend further credence to this hypothesis since the signal values are very close to the achiral system, indicating similar physical origin of both phenomena. This is not surprising since both achiral and chiral channels are synthesized using almost identical chemical processes (Supporting Information, Section I). Thus, the intrinsic asymmetries of the achiral channel remain in the case of the chiral channel as well, which results in very similar background responses. The effect of chirality manifests at certain angles. Next, it remains to be confirmed if the above phenomena persist even in the linear bias range or not.

To investigate the role of *transport nonlinearity*, we first measured the two-terminal current–voltage (*I–V*) characteristics of Fmoc-GG functionalized graphene samples using Au–Au electrodes for θ = 0° at *B* = ± 12 kG as shown in Figure 3(a). The average response from ∼ 15 scans from a given device are shown, with the error bars indicating the standard deviation. Reproducibility of these measurements are evident from the small error bars (∼0.15% of the measured current). This is an order of magnitude smaller than the magnetocurrent signal Δ*I* = *I*(+12 kG) – *I*(−12 kG), which is ∼ 2% of the measured current. The *I–V* curves are clearly nonlinear in the bias range of ± 1 V and the MR is evident from the *insets*. The MR measurements discussed in Figures 1 and 2 were performed at a bias of 0.5*V*. It is clear from Figure 3(a) that these measurements correspond to the *nonlinear* transport regime.

**Figure 3:**
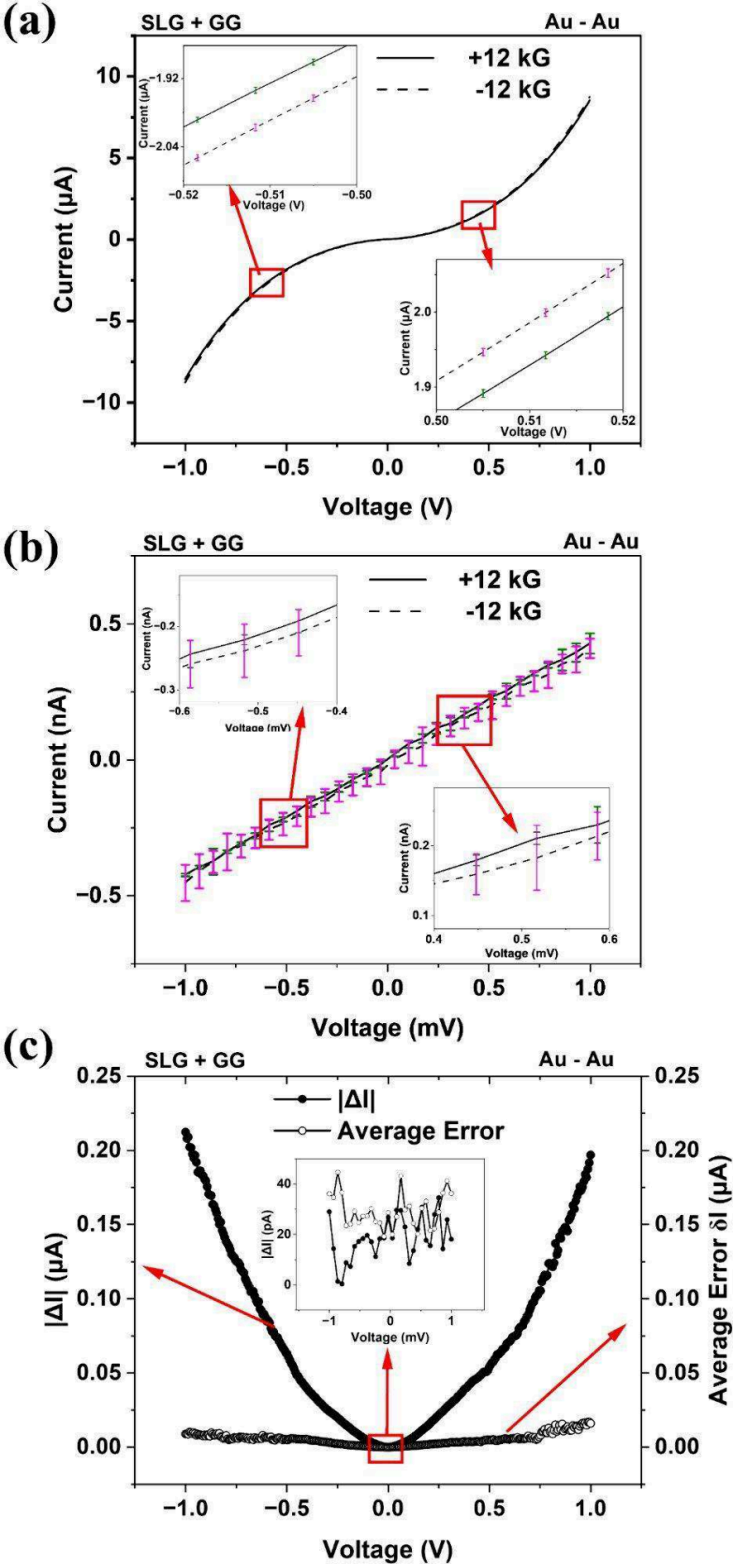
Current–voltage (*I–V*) characteristics of achiral SLG-GG samples at θ = 0°, using Au–Au electrodes over (a) nonlinear bias range and (b) linear bias range. The *I–V* scans represent the response averaged over >15 scans. Corresponding standard deviations (representing intrinsic noise of the samples) are shown using error bars. MR effect is observed in the nonlinear range but not in the linear range. Linear-range zero-bias conductances for ±12 kG have been calculated in Figure S7a,b, which are nominally identical. (c) Bias (*V*) dependence of |Δ*I*| = |*I*(+12 kG, *V*) – *I*(−12 kG, *V*)| and the “average error” *δI* (due to intrinsic sample noise), as discussed in the text.

Next, we investigate the *linear response* regime. Linear response refers to small ‘‘perturbation’’ of the equilibrium by a small voltage *V*, so that the current response is linear with respect to the applied voltage.^25^ While the true extent of the linear range is hard to ascertain, we adopt the conservative estimate of *V* < *k*_B_ *T/q* (∼ 0.78 mV in our case), which is a sufficient condition for linearity.^25^ This range of bias is difficult to probe using chiral insulators because of vanishingly small current but can be done relatively easily with more conductive samples such as ours.

Figure 3(b) shows the *I–V* characteristics, averaged over >15 scans, at ± 12 kG in the bias range of ± 0.78 mV. The linear response is evident, and significant overlap of the error bars are observed between ± 12 kG scans. The *instrumental error* in the linear range is ∼ 0.2% of the measured current, which is much smaller than the linear range error bars (∼2–16%) or even Δ*I* = *I*(+12 kG) – *I*(−12 kG), which is ∼ 2–10%. Thus, the error bars primarily represent the *intrinsic noise* of the chiral systems (scan-to-scan variation generated by random charge carrier movement or other stochastic processes inherent to the system), and *not* any instrumental error. Applying ± 12 kG in the linear range does not generate any systematic difference between the *I–V* curves beyond the intrinsic noise of the samples. Thus, these *I–V* curves are essentially identical, confirming the validity of Onsager’s relation. Figure S7­(a), (b) show linear-region conductance calculation at zero bias, and nominally identical values are found.

Figure 3(c) shows the bias (*V*) dependence of magnetocurrent |Δ*I*| (*V*) = |*I*(+12 kG, *V*) – *I*(−12 kG, *V*)| and *δI* (average of the error bars of the *I–V* scans or *intrinsic* noise of the samples). Clearly, |Δ*I*| falls below *δI* in the linear bias range (*inset*), and becomes more prominent as the bias is increased. Combined with the observations discussed above, it is evident that the *observed MR asymmetry is purely due to transport nonlinearity in asymmetric channels*, consistent with other systems reported in the past. We note that Δ*I* (*V*) is an odd function of *V* (Figure S3), consistent with past studies on the CISS effect.^26^

Ref 23 reported asymmetric MR in single wall carbon nanotubes in the nonlinear regime. The lowest order *B*-asymmetric component of the current was found to be symmetric (∼*V* ^2^) relative to applied bias *V*. While our observation of asymmetric MR in nanographene is consistent with this study, our *I–V* characteristics are antisymmetric with respect to *V* (Figure 3(a)). It has been pointed out that electron–electron or electron–phonon interactions are necessary for observation of CISS MR,^26,27^ in which case magnetocurrent Δ*I* is symmetric relative to *V* (i.e., ∼ *V* ^2^).^27^ Ref 26 has shown that Δ*I* is dominantly odd (cubic) with respect to *V* in the presence of spin–orbit interaction and strong Coulomb interactions. The system modeled in this work has a chiral geometry and one of the contacts is magnetic. Our results however indicate that Δ*I*, with odd bias dependence, can be observed even without those constraints.

To check whether the *chirality induced* MR component in Figures 2(d)-(f) is related to transport nonlinearity, we again investigate the *I–V* characteristics of these chiral films (with nonspin-detecting Au–Au electrodes) at θ = 90° in both linear and nonlinear range. Figure 4(a) shows the *I–V* characteristics of Fmoc-FF (D) functionalized SLG in the nonlinear range, where the MR manifests. At the bias of 0.5*V* used in the MR measurements in Figures 2(d)-(f) the *I–V*s are nonlinear. However, in the linear range (±0.78 mV) no MR is observed above the *intrinsic* noise level *of the samples* (Figure 4(b), (c)), consistent with Onsager’s reciprocity principle. Similar plots for Fmoc-FF (L) functionalized SLG are shown in Figure 5. Calculation of linear-region zero bias conductances for these samples are shown in Figure S7, confirming this result. Thus, *asymmetric MR mimicking the CISS effect can arise even for nonspin-detecting contacts* in the nonlinear transport regime. In some chiral molecular junctions *apparent* violation of Onsager’s reciprocity was reported,^28^ which can arise from an interfacial barrier that depends on chirality and *B*.^29,30^ However, in our case, transport is diffusive and our results are relatively independent of the contact metals (Au/Ni/Al), which are expected to form different barriers at the contacts. Thus, contact barriers do not play a critical role in our case and therefore, satisfaction of Onsager’s reciprocity is not unexpected.

**Figure 4:**
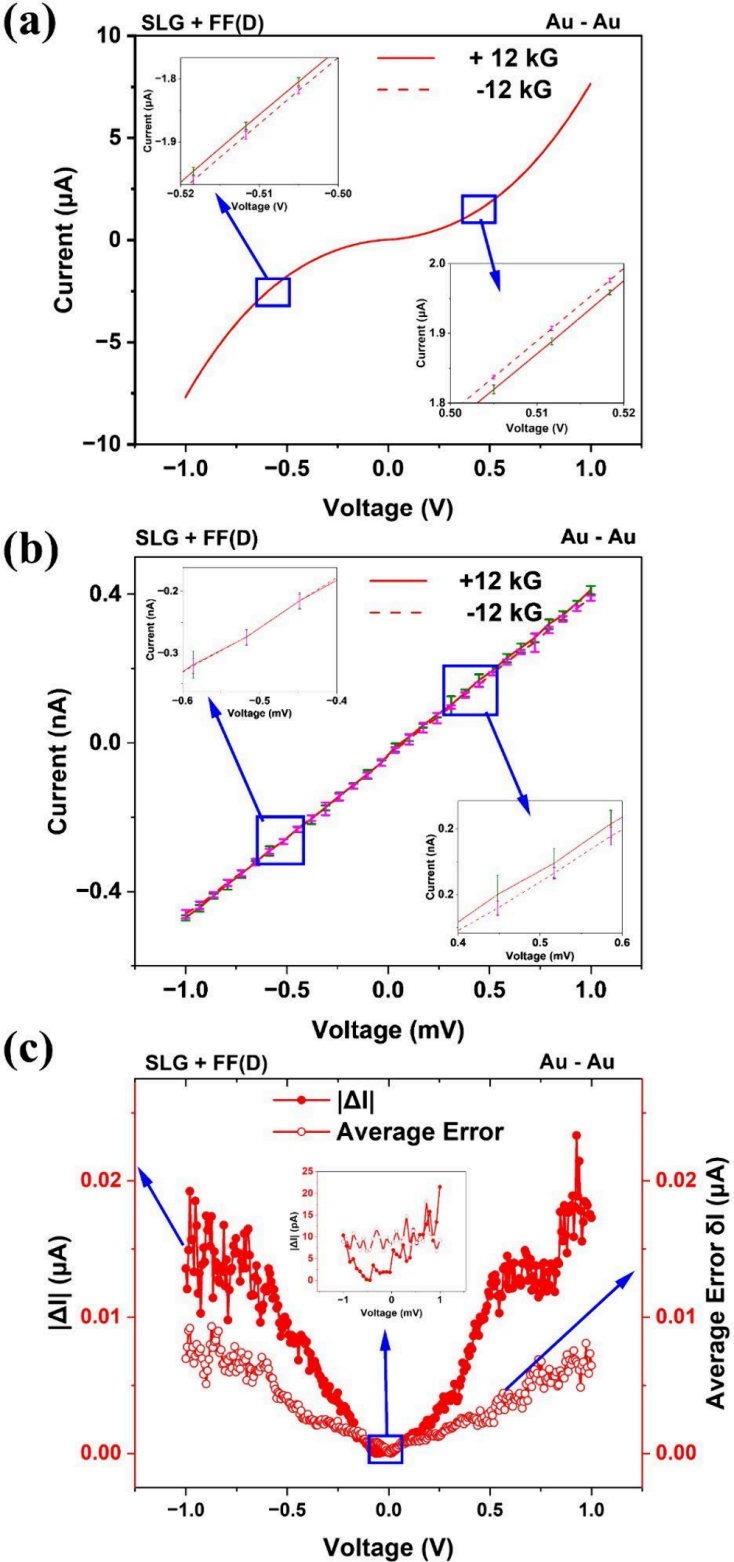
Current–voltage (*I–V*) characteristics of chiral SLG-Fmoc-FF­(D) samples at θ = 90°, using Au–Au electrodes over (a) nonlinear bias range and (b) linear bias range. The *I–V* scans represent the response averaged over >15 scans. Corresponding standard deviations (representing intrinsic noise of the samples) are shown using error bars. MR effect is observed in the nonlinear range but not in the linear range. Linear-range zero-bias conductances for ±12 kG have been calculated in Figure S7c,d, which are nominally identical. (c) Bias (*V*) dependence of |Δ*I*| = |*I*(+12 kG, *V*) – *I*(−12 kG, *V*)| and the “average error” *δI* (due to intrinsic sample noise), as discussed in the text.

**Figure 5:**
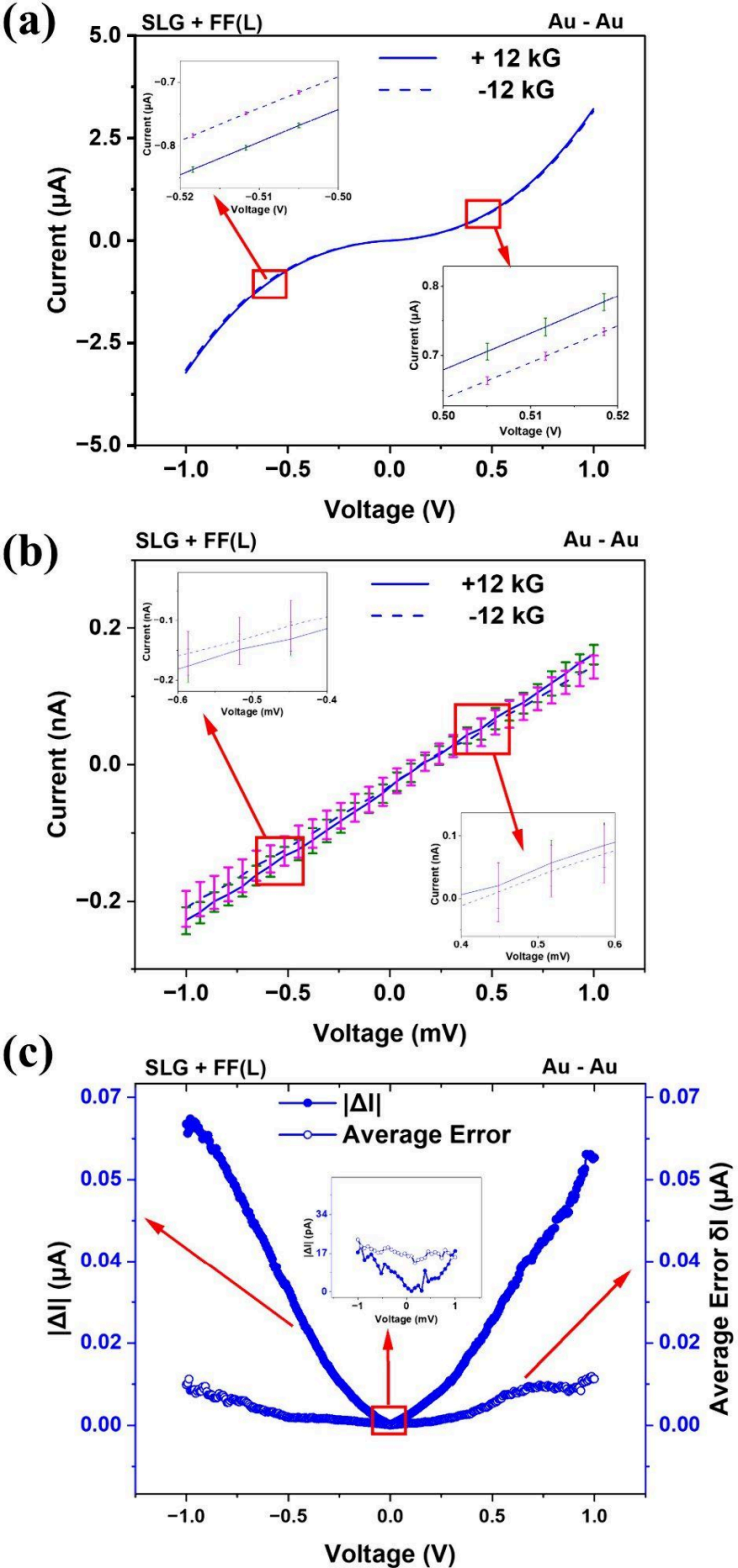
Current–voltage (*I–V*) characteristics of chiral SLG-Fmoc-FF­(L) samples at θ = 90°, using Au–Au electrodes over (a) nonlinear bias range and (b) linear bias range. The *I–V* scans represent the response averaged over >15 scans. Corresponding standard deviations (representing intrinsic noise of the samples) are shown using error bars. MR effect is observed in the nonlinear range but not in the linear range. Linear-range zero-bias conductances for ±12 kG have been calculated in Figure S7e,f, which are nominally identical. (c) Bias (*V*) dependence of |Δ*I*| = |*I*(+12 kG, *V*) – *I*(−12 kG, *V*)| and the “average error” *δI* (due to intrinsic sample noise), as discussed in the text.

Next, we compare our results with EMChA, which is also electrode-independent and is commonly observed in chiral conductors.^24,31^ In this case, for a fixed *B* and given chirality, a diode-like *I*(*V*) characteristics with *I*(+*V*) ≠ – *I*(−*V*) is observed. In our case, as seen from Figures 3(a), 4(a) and 5(a), *I*-*V* characteristics are antisymmetric in all cases i.e. *I*(+*V*) = – *I*(−*V*). Thus, the EMChA signal, quantified as [*R*(*B*, *I*) – *R*(*B*, – *I*)]/[*R*(*B*, *I*) + *R*(*B*, – *I*)],^24^ is negligible as shown in Figure S4. EMChA also shows linear *B* dependence of MR, whereas our Figure 1(c) and Figure S5 show a saturating behavior at higher magnetic fields, which is consistent with CISS MR.^4,13,32^ Thus, our results are not consistent with EMChA. Coexistence of CISS and EMChA has been reported in ref 33, where manifestation of EMChA was primarily attributed to large current densities (∼10^7^A/cm^2^) typical for single molecular junctions. For macroscopic devices such as ours, current density is orders of magnitude smaller (∼1 A/cm^2^), which may explain nonobservation of EMChA in the present case. In addition, our magnetic field range (∼1 T) is smaller than ref 33 (∼2 T).

Recently ref 34 reported a *chirality dependent MR* in chiral Au nanocrystals covered by an insulating shell, *even in the absence of any ferromagnetic electrode*. Compared to the present study, the MR in this case exhibits *different symmetries* relative to bias and magnetic field. This was explained based on the magnetic field dependence of the contact barrier, which is relevant to their core- (insulating) shell nanocrystals, but, as discussed earlier, not relevant in our case.

In summary, we have shown that asymmetric two-terminal MR is a general phenomenon in the nonlinear transport regime for asymmetric conductors and is *independent of the nature of the electrical contacts*. In addition, if the channel happens to be chiral, a chirality-dependent MR component is superimposed on the background asymmetric MR. This chiral MR component correlates with the supramolecular chirality of the system and shows the same symmetries of *I*(*V*) and *MR*(*B*) responses observed in other CISS systems, therefore satisfying all the conditions of CISS MR. Since nonlinear transport and channel asymmetry are common in almost all CISS systems, we expect the results to be of relevance in other chiral systems as well. Thus, observation of CISS MR may not necessarily be attributed to detection of chirality dependent spins.

## Supplementary Material



## Notes

Sample fabrication and additional analysis and characterizations (PDF)

## References

[1] Aiello C. D., Abendroth J. M., Abbas M., Afanasev A., Agarwal S., Banerjee A. S., Beratan D. N., Belling J. N., Berche B., Botana A., Caram J. R., Celardo G. L., Cuniberti G., Garcia-Etxarri A., Dianat A., Diez-Perez I., Guo Y., Gutierrez R., Herrmann C., Hihath J., Kale S., Kurian P., Lai Y.-C., Liu T., Lopez A., Medina E., Mujica V., Naaman R., Noormandipour M., Palma J. L., Paltiel Y., Petuskey W., Ribeiro-Silva J. C., Saenz J. J., Santos E. J. G., Solyanik-Gorgone M., Sorger V. J., Stemer D. M., Ugalde J. M., Valdes-Curiel A., Varela S., Waldeck D. H., Wasielewski M. R., Weiss P. S., Zacharias H., Wang Q. H. (2022). A Chirality-Based
Quantum Leap. ACS Nano.

[2] Bloom B. P., Paltiel Y., Naaman R., Waldeck D. H. (2024). Chiral Induced Spin
Selectivity. Chem. Rev..

[3] Firouzeh S., Hossain M. A., Cuerva J. M., Álvarez de Cienfuegos L., Pramanik S. (2024). Chirality-Induced Spin
Selectivity in Composite Materials:
A Device Perspective. Acc. Chem. Res..

[4] Kiran V., Mathew S. P., Cohen S. R., Delgado I. H., Lacour J., Naaman R. (2016). Helicenes—A New Class of Organic Spin Filter. Adv. Mater..

[5] Mishra S., Mondal A. K., Smolinsky E. Z. B., Naaman R., Maeda K., Nishimura T., Taniguchi T., Yoshida T., Takayama K., Yashima E. (2020). Spin Filtering
Along Chiral Polymers. Angew. Chem..

[6] Mondal A. K., Preuss M. D., Ślęczkowski M. L., Das T. K., Vantomme G., Meijer E. W., Naaman R. (2021). Spin Filtering in Supramolecular
Polymers Assembled from Achiral Monomers Mediated by Chiral Solvents. J. Am. Chem. Soc..

[7] Rahman Md. W., Firouzeh S., Mujica V., Pramanik S. (2020). Carrier Transport Engineering
in Carbon Nanotubes by Chirality-Induced Spin Polarization. ACS Nano.

[8] Rahman Md. W., Mañas-Torres M. C., Firouzeh S., Cuerva J. M., Álvarez de Cienfuegos L., Pramanik S. (2021). Molecular Functionalization
and Emergence of Long-Range Spin-Dependent Phenomena in Two-Dimensional
Carbon Nanotube Networks. ACS Nano.

[9] Rahman Md. W., Alam K. M., Pramanik S. (2018). Long Carbon Nanotubes Functionalized
with DNA and Implications for Spintronics. ACS
Omega.

[10] Hossain M. A., Illescas-Lopez S., Nair R., Manuel Cuerva J., Álvarez de Cienfuegos L., Pramanik S. (2023). Transverse Magnetoconductance
in Two-Terminal Chiral Spin-Selective Devices. Nanoscale Horizons.

[11] Wang C., Liang Z.-R., Chen X.-F., Guo A.-M., Ji G., Sun Q.-F., Yan Y. (2024). Transverse Spin Selectivity in Helical
Nanofibers Prepared without Any Chiral Molecule. Phys. Rev. Lett..

[12] Rahman Md. W., Mañas-Torres M. C., Firouzeh S., Illescas-Lopez S., Cuerva J. M., Lopez-Lopez M. T., Álvarez de Cienfuegos L., Pramanik S. (2022). Chirality-Induced Spin
Selectivity in Heterochiral
Short-Peptide-Carbon-Nanotube Hybrid Networks: Role of Supramolecular
Chirality. ACS Nano.

[13] Kulkarni C., Mondal A. K., Das T. K., Grinbom G., Tassinari F., Mabesoone M. F. J., Meijer E. W., Naaman R. (2020). Highly Efficient and
Tunable Filtering of Electrons’ Spin by Supramolecular Chirality
of Nanofiber-Based Materials. Adv. Mater..

[14] Firouzeh S., Illescas-Lopez S., Hossain M. A., Cuerva J. M., Álvarez
de Cienfuegos L., Pramanik S. (2023). Chirality-Induced Spin Selectivity
in Functionalized Carbon Nanotube Networks: The Role of Spin-Orbit
Coupling. J. Chem. Phys..

[15] Firouzeh S., Illescas-Lopez S., Hossain M. A., Cuerva J. M., Álvarez
de Cienfuegos L., Pramanik S. (2023). Chirality-Induced Spin Selectivity
in Supramolecular Chirally Functionalized Graphene. ACS Nano.

[16] Shlimak I., Zion E., Butenko A. V., Wolfson L., Richter V., Kaganovskii Yu., Sharoni A., Haran A., Naveh D., Kogan E., Kaveh M. (2016). Hopping Magnetoresistance in Ion
Irradiated Monolayer Graphene. Physica E: Low-dimensional
Systems and Nanostructures.

[17] Lu H., Wang J., Xiao C., Pan X., Chen X., Brunecky R., Berry J. J., Zhu K., Beard M. C., Vardeny Z. V. (2019). Spin-Dependent Charge Transport through 2D Chiral Hybrid
Lead-Iodide Perovskites. Science Advances.

[18] Adhikari Y., Liu T., Wang H., Hua Z., Liu H., Lochner E., Schlottmann P., Yan B., Zhao J., Xiong P. (2023). Interplay
of Structural Chirality, Electron Spin and Topological Orbital in
Chiral Molecular Spin Valves. Nat. Commun..

[19] Liu Y., Xiao J., Koo J., Yan B. (2021). Chirality-Driven Topological
Electronic Structure of DNA-like Materials. Nat. Mater..

[20] Brandenstein-Köth B., Worschech L., Forchel A. (2010). Magnetic-Field Asymmetry of Nonlinear
Mesoscopic Transport in Channels Coupled to a Single Metallic Gate. Physica E: Low-dimensional Systems and Nanostructures.

[21] Guo A.-M., Pan T.-R., Fang T.-F., Xie X. C., Sun Q.-F. (2016). Spin Selectivity
Effect in Achiral Molecular Systems. Phys. Rev.
B.

[22] Sánchez D., Büttiker M. (2004). Magnetic-Field Asymmetry of Nonlinear Mesoscopic Transport. Phys. Rev. Lett..

[23] Wei J., Shimogawa M., Wang Z., Radu I., Dormaier R., Cobden D. H. (2005). Magnetic-Field Asymmetry of Nonlinear Transport in
Carbon Nanotubes. Phys. Rev. Lett..

[24] Rikken G. L. J. A., Avarvari N. (2019). Strong Electrical Magnetochiral
Anisotropy in Tellurium. Phys. Rev. B.

[25] Datta, S. Electronic Transport in Mesoscopic Systems; Cambridge Studies in Semiconductor Physics and Microelectronic Engineering; Cambridge University Press: Cambridge, 1995. 10.1017/CBO9780511805776.

[26] Huisman K. H., Heinisch J.-B. M.-Y., Thijssen J. M. (2023). Chirality-Induced
Spin Selectivity
(CISS) Effect: Magnetocurrent-Voltage Characteristics with Coulomb
Interactions I. J. Phys. Chem. C.

[27] Huisman K. H., Thijssen J. M. (2021). CISS Effect: A Magnetoresistance Through Inelastic
Scattering. J. Phys. Chem. C.

[28] Liu T., Wang X., Wang H., Shi G., Gao F., Feng H., Deng H., Hu L., Lochner E., Schlottmann P., von Molnár S., Li Y., Zhao J., Xiong P. (2020). Linear and Nonlinear Two-Terminal
Spin-Valve Effect from Chirality-Induced
Spin Selectivity. ACS Nano.

[29] Tirion S. H., van Wees B. J. (2024). Mechanism for Electrostatically
Generated Magnetoresistance
in Chiral Systems without Spin-Dependent Transport. ACS Nano.

[30] Zhao Y., Zhang K., Xiao J., Sun K., Yan B. (2025). Magnetochiral
Charge Pumping Due to Charge Trapping and Skin Effect in Chirality-Induced
Spin Selectivity. Nat. Commun..

[31] Rikken G. L. J. A., Avarvari N. (2023). Comparing Electrical
Magnetochiral Anisotropy and Chirality-Induced
Spin Selectivity. J. Phys. Chem. Lett..

[32] Das T. K., Naaman R., Fransson J. (2024). Insights into the Mechanism of Chiral-Induced
Spin Selectivity: The Effect of Magnetic Field Direction and Temperature. Adv. Mater..

[33] Singh A.-K., Martin K., Mastropasqua Talamo M., Houssin A., Vanthuyne N., Avarvari N., Tal O. (2025). Single-Molecule Junctions Map the
Interplay between Electrons and Chirality. Nat.
Commun..

[34] Wu F., Wang Y., Zhao Y., Yang Z., Tian Y., Xie Z., Niu W., Yan B., Guo C. (2025). Enantiomer-Selective
Magnetoresistance in Chiral Gold Nanocrystals by Magnetic Control
of Surface Potentials. arXiv.

