# Supplementary material for: Observation of Two-Terminal CISS Magnetoresistance with Nonmagnetic Contacts
Source: Nano Lett. 2025 Jun 9;25(24):9623–30. doi: 10.1021/acs.nanolett.5c01297 (PMC12186623; doi:10.1021/acs.nanolett.5c01297)
Supplement: Supplementary file 1 [file nl5c01297_si_001.pdf]

Supporting Information

Observation of Two-terminal CISS Magnetoresistance with Non-magnetic Contacts

Md Anik Hossain,[a,†] Sara Illescas-Lopez,[b] Seyedamin Firouzeh,[a,†] Juan Manuel Cuerva,[b] Luis Álvarez de Cienfuegos,[b,c,*] Sandipan Pramanik[a,*]

[a] Department of Electrical and Computer Engineering, University of Alberta, Edmonton, Alberta T6G 1H9, Canada
[b] Universidad de Granada, Departamento de Química Orgánica, Unidad de Excelencia Química Aplicada a Biomedicina y Medioambiente, C. U. Fuentenueva, Avda. Severo Ochoa s/n, E-18071 Granada, Spain
[c] Instituto de Investigación Biosanitaria ibs. Avda. De Madrid, 15, E-18016 Granada, Spain

† Equal contributions.
*Corresponding Authors: lac@ugr.es, spramani@ualberta.ca

I. Sample fabrication.

N-Fluorenylmethoxycarbonyl-L-diphenylalanine (Fmoc-FF(L)) and N-Fluorenylmethoxycarbonyl-D-diphenylalanine (Fmoc-FF (D)) were purchased from LifeTein, USA. N-Fluoroenylmethoxycarbonyl-diglycine (Fmoc-GG) was purchased from Fluorochem, UK. All Fmoc-peptides were used without further purification. SLG samples were purchased from Alfa Chemistry (CAS No.: 7782-42-5), prepared by thermal exfoliation reduction and hydrogen reduction of graphite oxide (GO), consist of 1–5 atomic layers of graphene with typical flake size ~ 0.5–5 μm. The oxygen content is estimated to be ~7–7.5%.

To obtain Fmoc-peptides+SLG composites, basic solutions of Fmoc-FF(L/D) and Fmoc-GG were first prepared. Fmoc-FF(L/D) and Fmoc-GG peptides were weighed separately into a vial and deionized water was added to obtain a final concentration of 10 mM. The suspension was sonicated (HSt Powersonic 405 ultrasonic bath) for 1 hour. Then, a NaOH solution (0.5 M) was added dropwise until a clear solution was obtained (pH=10.7). The pH was measured using a HACH sensor PH 3 pH meter. The pH meter was calibrated using pH 4, pH 7 and pH 10 buffer solutions.

To prepare the graphene flakes peptide solution, 0.7 mg of graphene flakes were separately weighed into a vial tube. The graphene flakes were suspended in 1 mL of the basic aqueous solution of: Fmoc-FF(L/D) or Fmoc-GG (prepared above). The suspension was sonicated for 2.5 hours in a cold ultrasonic bath and then centrifuged for 5 minutes at 10,000 rpm (Sigma 1-14 centrifuge). Finally, the supernatant was carefully collected.

Final hydrogels were obtained using glucono-δ-lactone (GDL) or sodium carbonate (Na_2CO_3) as a gelling agent. In the case of GDL, 2 equivalents of GDL were added to the composite solutions and mixed by vortexing. For Na_2CO_3 hydrogels a final concentration of 25 mM sodium carbonate was added to the basic composite solution and mixed by vortexing. After 12 hours, both types of hydrogels were completely formed.

Typical contact thickness is ~100 nm in all cases, and the gap between the contacts is ~ 2μm. The graphene film is drop-cast on the contacts from a gel solution and subsequently annealed. Due to the relatively shallow height of the contact walls compared to the contact gap, the drop-cast film is expected to fill the gap completely.

II. CD Characterization of functionalized samples.

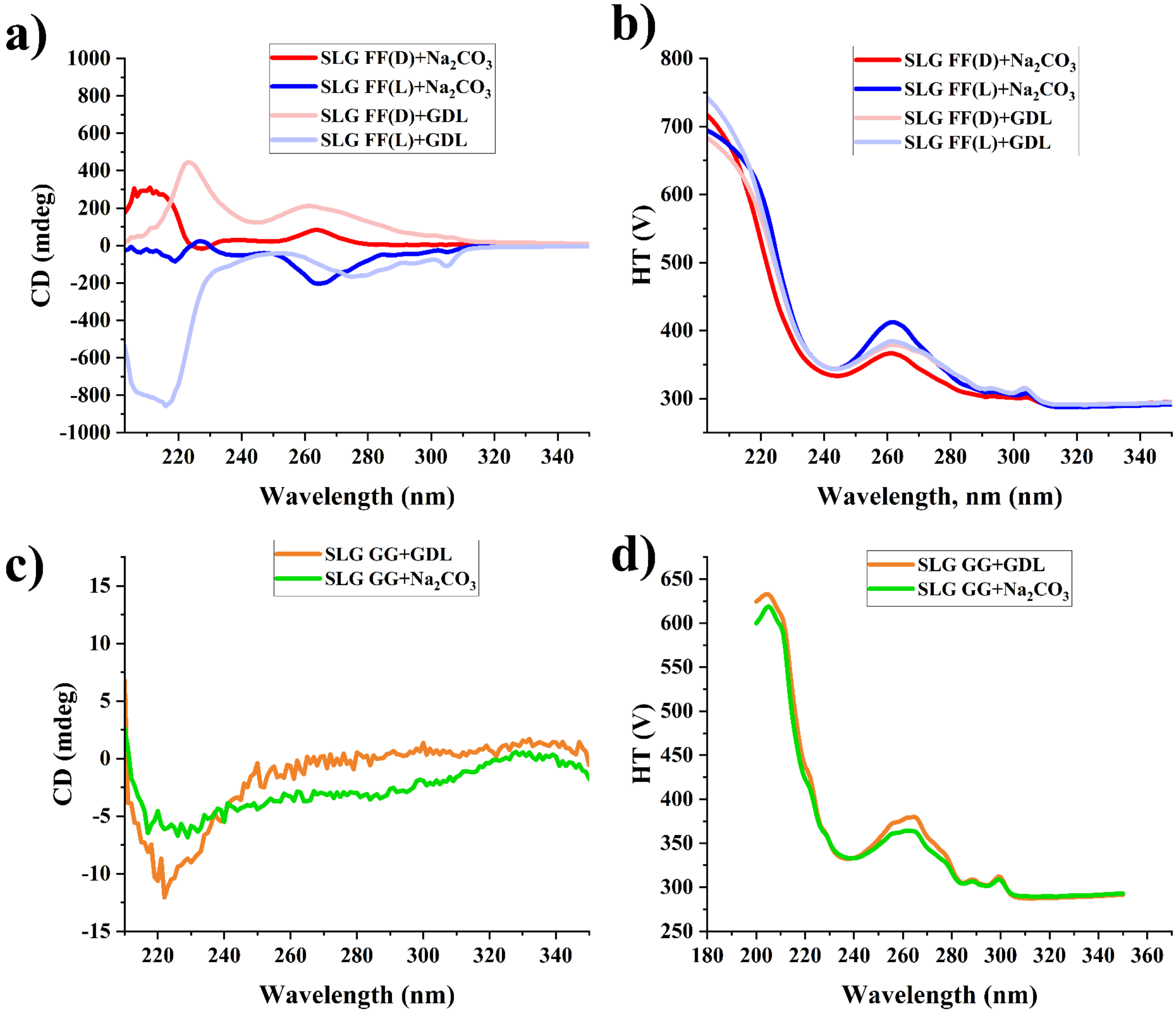

Figure S1: (a), (c) CD characterizations of SLG functionalized with Fmoc-FF(L/D) and Fmoc-GG respectively. Two different gelling agents were used in each case – achiral Na_2CO_3 and chiral GDL. Regardless, chirality of the final structure remains the same. (b), (d) HT spectra of SLG functionalized with Fmoc-FF(L/D) and Fmoc-GG respectively.

III. MR asymmetry with Al-Al contacts.

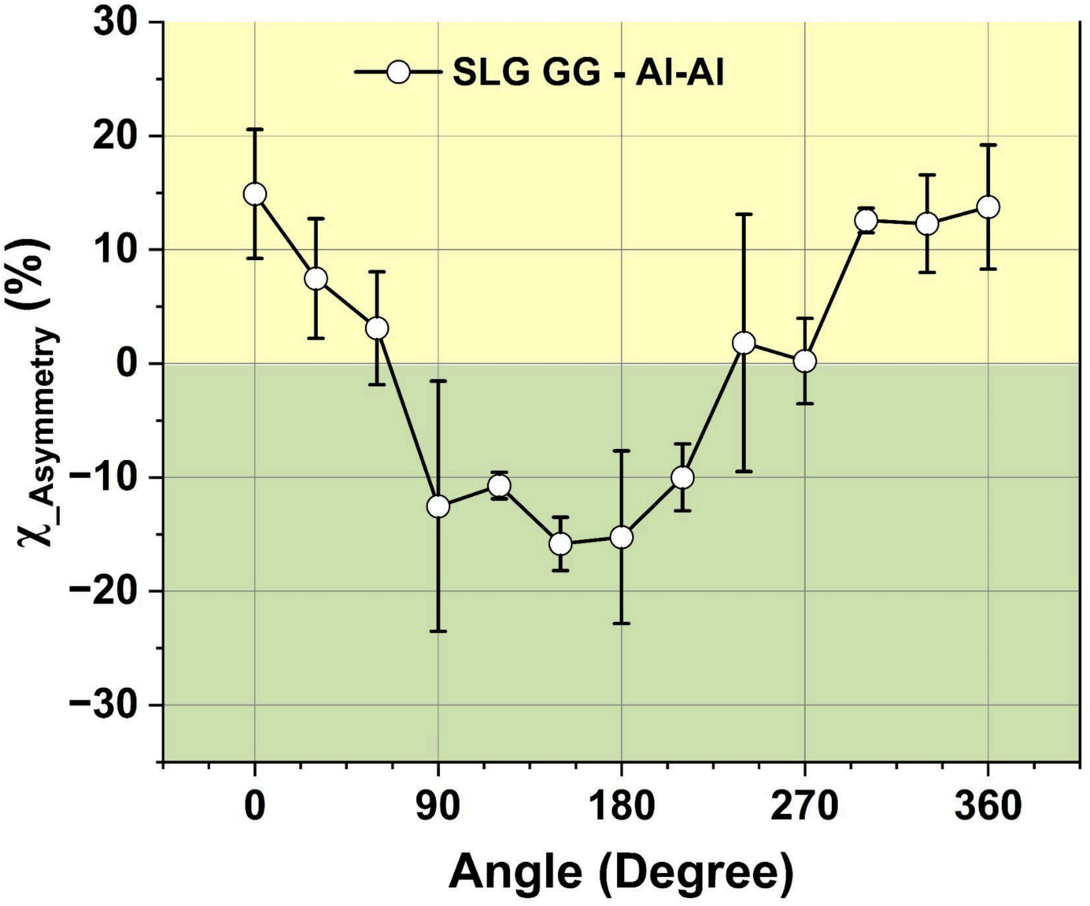

Figure S2. Fmoc-GG functionalized graphene samples with Al-Al contacts. MR results are qualitatively similar to those observed in Figures 2(a), (b).

IV. Bias dependence of magnetocurrent.

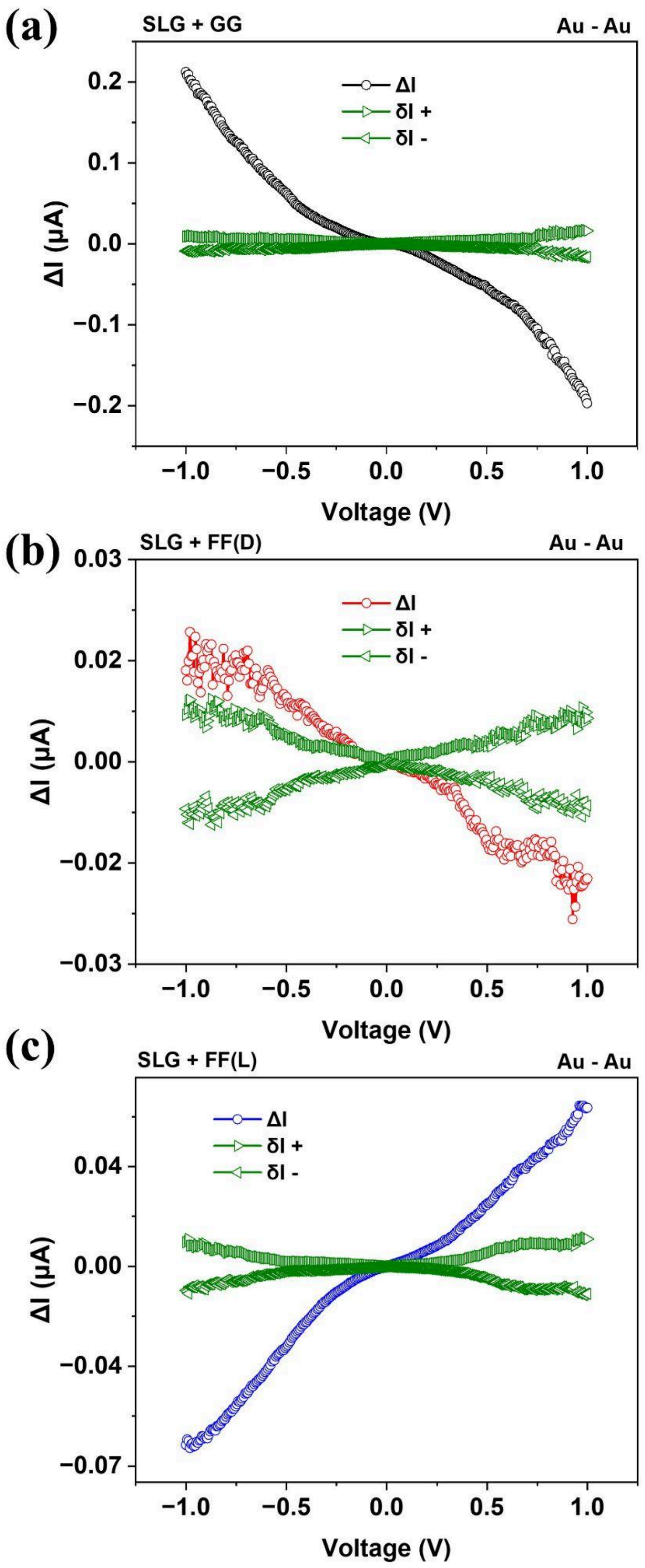

Figure S3. Odd bias dependence of the magnetocurrent ΔI (V) = I(+12kG, V) – I(–12kG, V) for (a) Fmoc-GG, (b) Fmoc-FF(D) and (c) Fmoc-FF(L) functionalized SLG samples. For Figure (a), the measurement angle θ = 0º, whereas for Figures (b) and (c), θ = 90º. Chirality dependence of the magnetocurrent is evident from Figures (b), (c). In all cases the average error $\pm\delta I$ is shown, which represents *intrinsic noise* of the chiral system as discussed in the paper..

V. Comparison to EMChA – Bias dependence.

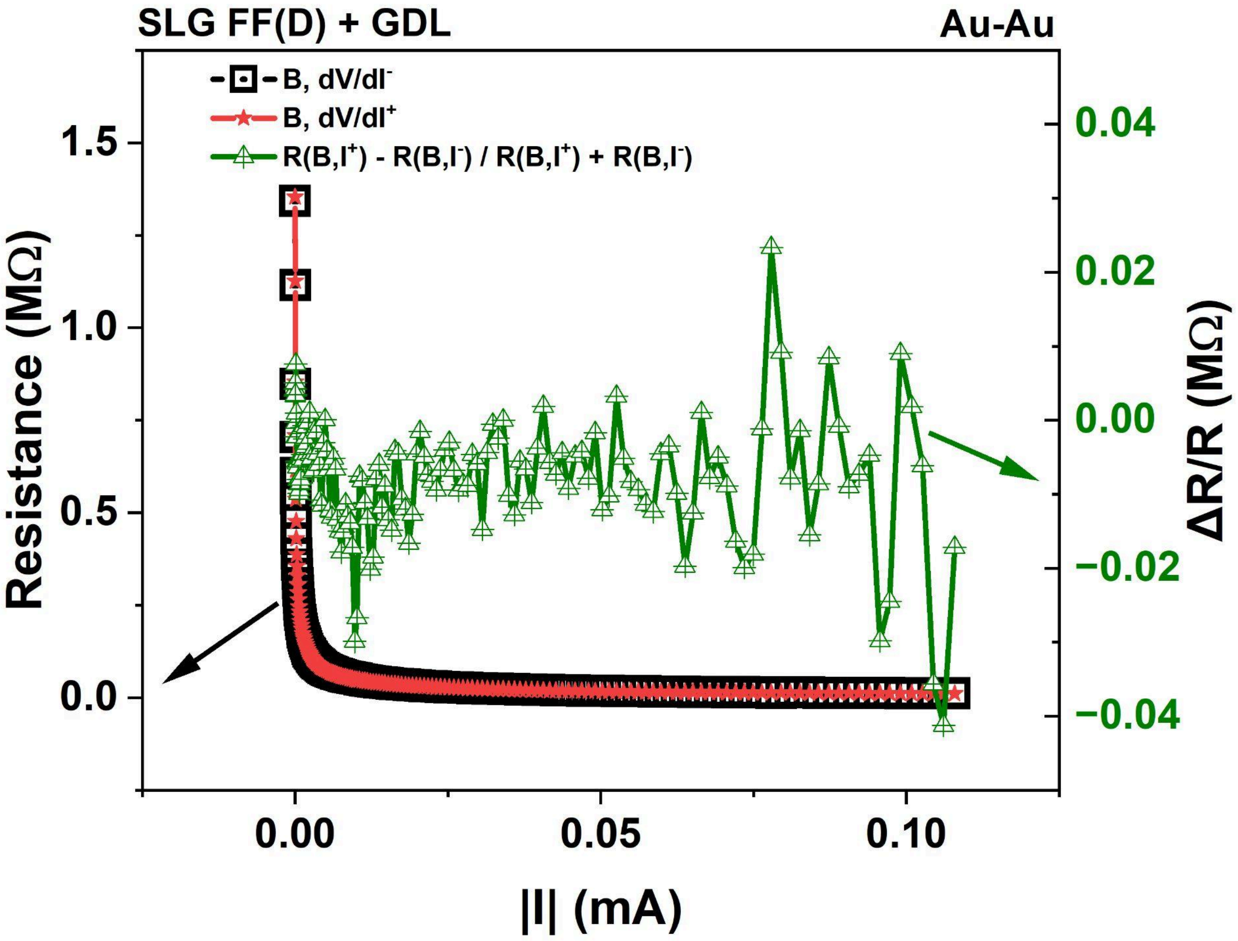

Figure S4. Plot of device resistance (R = dV/dI) computed for positive current I^+ (red curve) and negative current I^- (black curve) at a fixed B = 12 kG. The resistance values are almost identical for a given *magnitude* of current, irrespective of polarity. Thus, the *EMChA signal*, defined as $[R(B,I\ ) - R(B,-I)]\ /\ [R(B,I\ ) + R(B,-I)] \equiv \Delta R/R$ is almost negligible (green curve).

VI. Comparison to EMChA – *B* dependence.

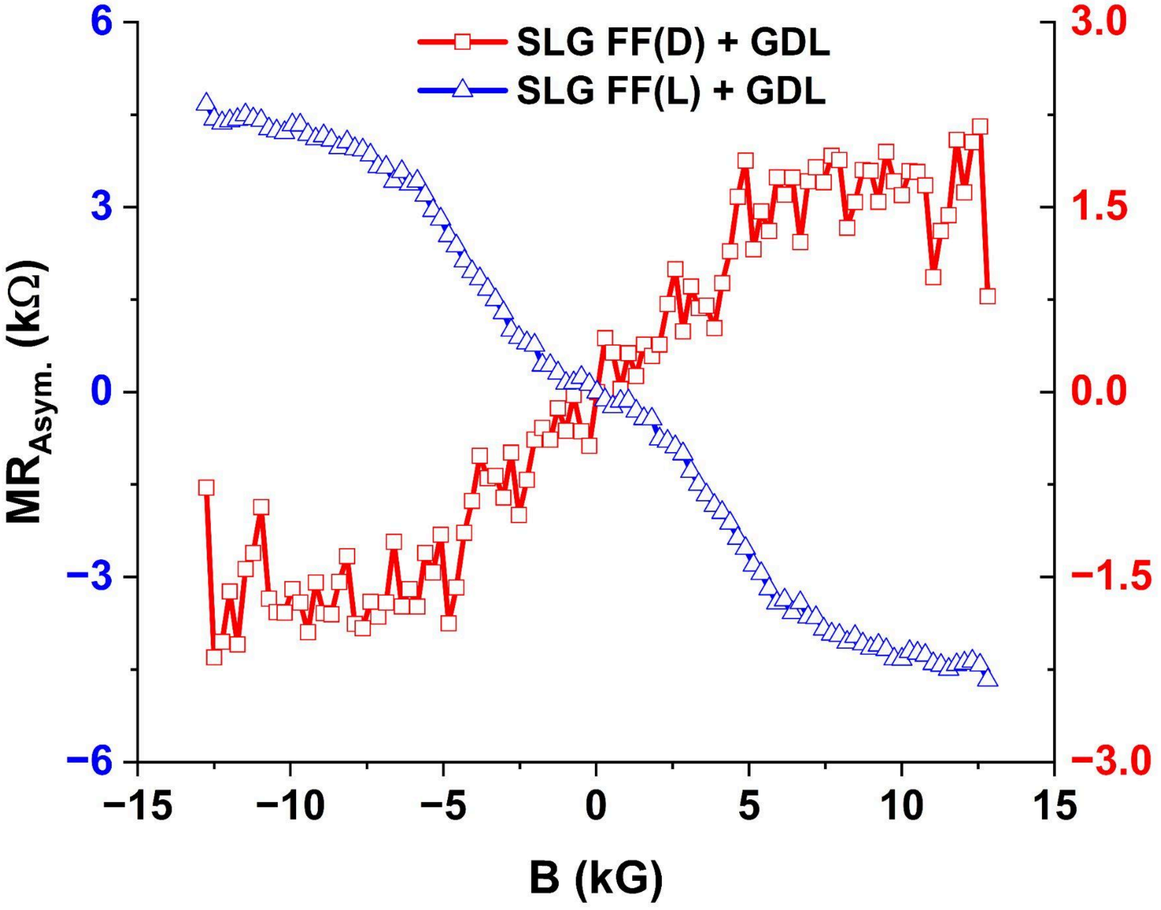

Figure S5. Plot of MR_{Asym} (B) for D and L functionalized samples for θ = 90° and 0.5V bias. The MR signal saturates at higher B, which is not consistent with EMChA. Such saturating behavior, however, is consistent with CISS systems reported earlier.

VII. Components of $\chi_{\text{Asym.}}$

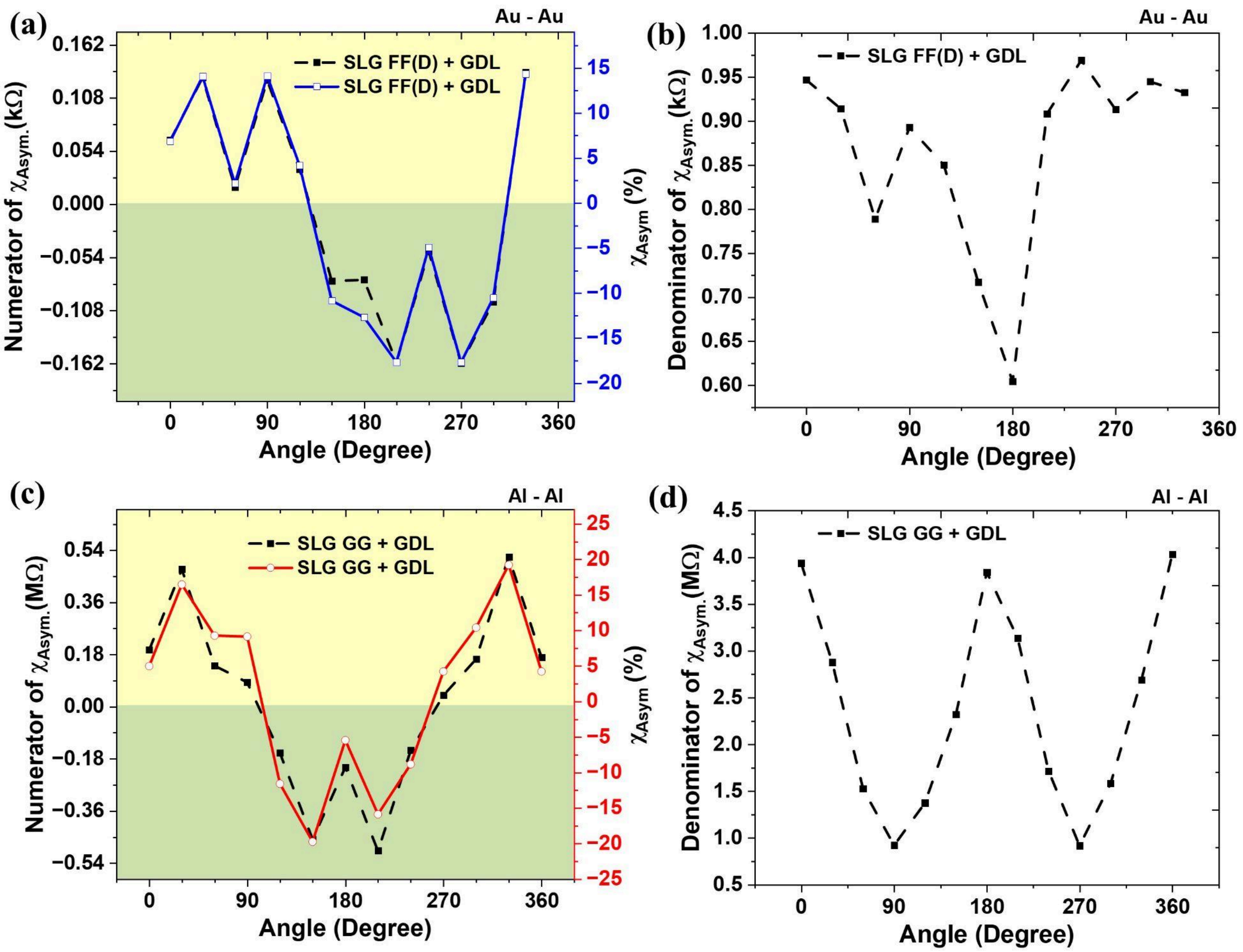

Figure S6. Numerator [MR_{Asym}(+12 kG) – MR_{Asym} (–12 kG)] and denominator [MR_{Sym} (0 kG) – MR_{Sym} (±12 kG)] of χ_{Asym} and their angular (θ) dependence. As seen from Figures (a), (c), the behavior of χ_{Asym} matches closely with the numerator. Responses from two different samples are shown – (a), (b) SLG + Fmoc-FF(D) + GDL and (c), (d) SLG + Fmoc-GG + GDL.

VIII. Calculation of I-V slope in the linear response range.

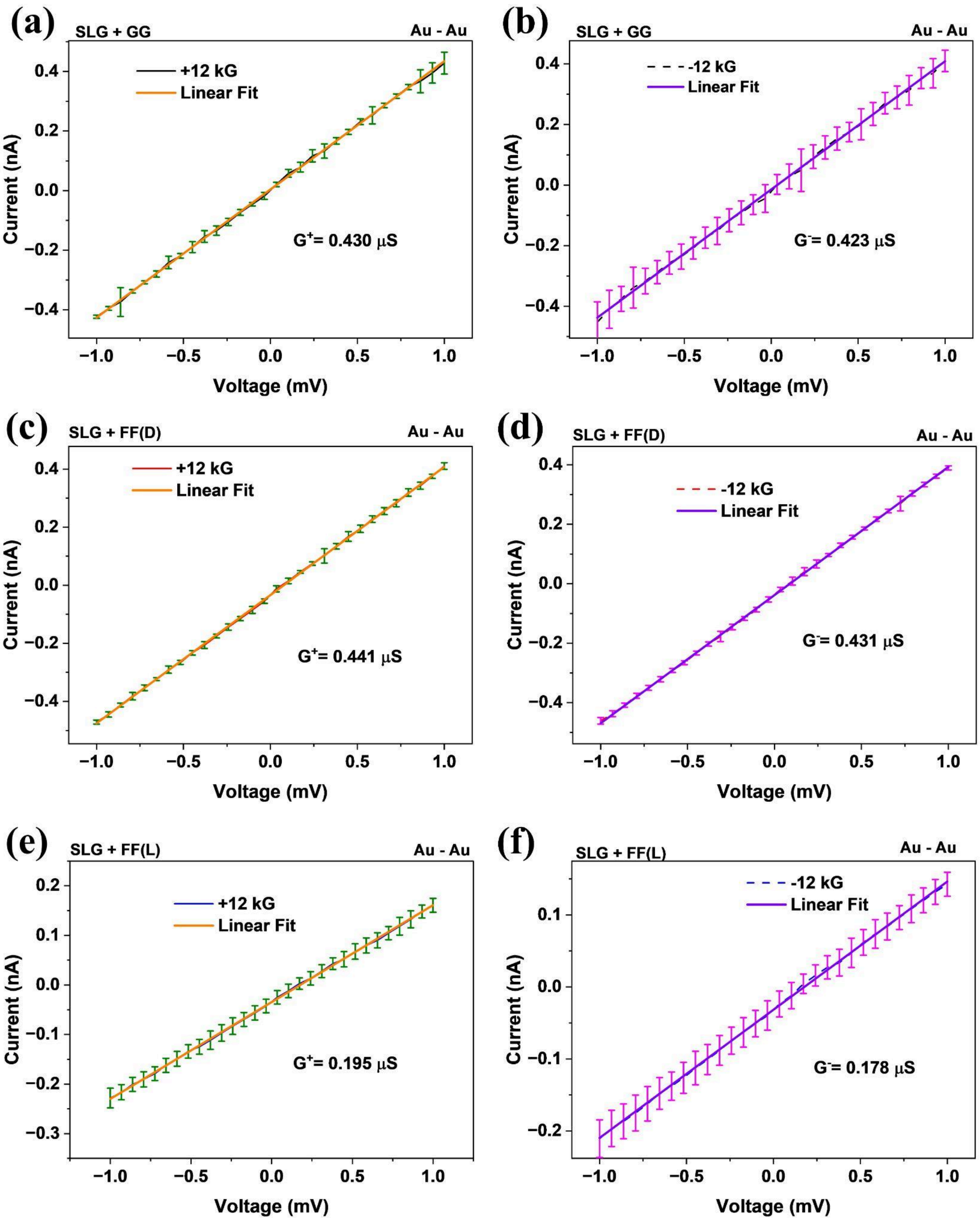

Figure S7. Calculation of the linear-region slopes of the *I-V* curves (or, zero-bias conductance) for +12 kG and –12kG for (a), (b) SLG+GG; (c), (d) SLG+FF(D) and (e), (f) SLG+FF(L). As shown in **Table 1** below, the difference in slopes (ΔG) is lower or of the order of the intrinsic noise level (δG).

Table 1.

	G^+	G^-	ΔG	δG (±)
**SLG +GG**	0.430 μS	0.423 μS	0.007 μS	0.066 μS
**SLG + FF(D)**	0.441 μS	0.431 μS	0.010 μS	0.010 μS
**SLG + FF(L)**	0.195 μS	0.178 μS	0.017 μS	0.026 μS

Here $\Delta G = G^+ - G^-$, as determined from the linear fits of the *I-V* curves. G^+ is the conductance calculated from the fitted linear slope of *I*(+12 kG), and G^- is the conductance calculated from the fitted linear slope of *I*(-12 kG). To calculate the intrinsic noise (δG) in the slope calculation, we consider SD_{IB+} and SD_{IB-} which are the standard deviations of the *I-V* curves (Figure 3b, 4b and 5b) for +12 kG and –12 kG, respectively, evaluated at *Bias Voltage* = 1mV . Next, δG is calculated using the following formula:

$$\delta G = \frac{\sqrt{SD_{I_{B+}}^2 + SD_{I_{B-}}^2}}{Bias\ Voltage}$$

As can be seen from the table, ΔG is lower or of the order of the intrinsic noise level δG, implying that the slopes are essentially identical for +12kG and –12kG in the linear range, satisfying Onsager's reciprocity.
